# Detection of bladder, breast and prostate cancer using serum and tissue miRNA profiling

**DOI:** 10.1186/gb-2011-12-s1-p18

**Published:** 2011-09-19

**Authors:** Patricia Porter-Gill, Yi-Ping Fu, Alpana Kaushiva, Douglas Price, William Dahut, William Figg, Ludmila Prokunina-Olsson

**Affiliations:** 1 Laboratory of Translational Genomics, Division of Cancer Epidemiology and Genetics, NCI/NIH, Bethesda, 20892, USA; 2 Molecular Oncology Branch, NCI/NIH, Bethesda, 20892, USA

## Background

miRNAs are short, non-coding regulatory RNA molecules that can bind to complementary sequences on target mRNAs, resulting in translational repression and gene silencing. miRNAs are attractive as biomarkers, because they are stable in various conditions and are easy to measure using quantitative PCR methods. Biomarkers that can differentiate between normal and tumor states and can be measured in easily accessible body fluids, such as blood and urine, are important for cancer diagnostics and disease monitoring.

## Methods and results

In this study, we identified a universal panel of miRNAs for cancer detection. This panel can easily be used to screen the serum of healthy individuals and patients with different types of cancer. First, we measured the expression of about 800 miRNAs in 40 control individuals and 60 patients with bladder, breast or prostate cancer using TaqMan Low Density gene expression arrays (Applied Biosystems), starting from 250 μl serum. On the basis of these results, we selected a panel of 24 miRNAs that showed the best discrimination between normal samples and cancer samples. These miRNAs were then retested as a custom-designed mini-panel on serum samples from 44 healthy controls and from patients with cancer (31 with bladder cancer, 25 with breast cancer and 28 with prostate cancer), as well as in relevant normal and tumor tissue samples (42 normal bladder samples and 43 bladder tumors, 44 normal breast samples and 42 breast tumors, and 50 normal prostate samples and 20 prostate tumors). Only miRNAs with changes in expression in the same direction in serum and tissue samples and with a significant association with cancer in both sample types were used for further analysis.

The current panel consists of 16 miRNAs: 15 targets and 1 positive control. Using this panel on serum samples from 77 controls, 52 patients with bladder cancer, 48 patients with breast cancer and 34 patients with prostate cancer, we performed receiver operating characteristic (ROC) analysis and achieved complete discrimination (area under the ROC curve (AUC) of about 1.0) between all types of cancers and controls, as well as good discrimination between different types of cancers (minimal AUC of 0.89 for breast and bladder cancer samples).

## Conclusions

Our results prove that miRNA detection in serum might be a promising method for cancer detection.

